# Logical negation mapped onto the brain

**DOI:** 10.1007/s00429-019-01975-w

**Published:** 2019-11-04

**Authors:** Yosef Grodzinsky, Isabelle Deschamps, Peter Pieperhoff, Francesca Iannilli, Galit Agmon, Yonatan Loewenstein, Katrin Amunts

**Affiliations:** 1 Edmond and Lily Safra Center for Brain Research, Goodman Building, Safra Campus, The Hebrew University, 91904 Jerusalem, Israel; 2 Institute of Neuroscience and Medicine (INM-1), Forschungszentrum Jülich, Jülich, Germany; 3 Cervo Research Centre, Faculty of Medicine, Laval University, Quebec City, Canada; 4 Department of Neurobiology, The Alexander Silberman Institute of Life Sciences and The Federmann Center for the Study of Rationality, The Hebrew University, Jerusalem, Israel; 5 C. & O. Vogt Institute for Brain Research, University Hospital Düsseldorf, Heinrich Heine University Düsseldorf, Düsseldorf, Germany; 6 Faculty of Human Services and Community Safety, Georgian College, Orillia, Ontario Canada

**Keywords:** Language, Logic, Numerosity, Functional neuroanatomy, Functional neuroimaging, Cytoarchitecture, Brain mapping, Negation, Sentence verification, Left anterior insula, Modularity

## Abstract

High-level cognitive capacities that serve communication, reasoning, and calculation are essential for finding our way in the world. But whether and to what extent these complex behaviors share the same neuronal substrate are still unresolved questions. The present study separated the aspects of logic from language and numerosity—mental faculties whose distinctness has been debated for centuries—and identified a new cytoarchitectonic area as correlate for an operation involving logical negation. A novel experimental paradigm that was implemented here in an RT/fMRI study showed a single cluster of activity that pertains to logical negation. It was distinct from clusters that were activated by numerical comparison and from the traditional language regions. The localization of this cluster was described by a newly identified cytoarchitectonic area in the left anterior insula, ventro-medial to Broca’s region. We provide evidence for the congruence between the histologically and functionally defined regions on multiple measures. Its position in the left anterior insula suggests that it functions as a mediator between language and reasoning areas.

**Electronic supplementary material:**

The online version of this article (10.1007/s00429-019-01975-w) contains supplementary material, which is available to authorized users.

## Introduction

The relation between linguistic, logical, and numerical abilities has long been debated. All three use formal rules to string smaller meaning-bearing forms into bigger ones, in a manner that ensures rich expressiveness. The thought that these emanate from one and the same neurocognitive system may thus be natural. Indeed, Aristotle believed that language and logic go hand in hand, as did the seventieth century *Port Royal* logicians/grammarians (cf. Gabbay et al. 2006; Chomsky and McGilvray 2009 for a historical perspective). Many modern linguists likewise believe that language and logic are interwoven in the human mind, which hosts a “natural logic…, a theory about the logical structure of natural language sentences and the regularities governing the notion of a valid argument for reasoning in natural language” (Lakoff 1970). But prominent philosophers in the modern era, notably Frege and Russell, thought otherwise and argued for their distinctness (van Heijenoort 1967). Many among these logicians focused on logic as a vehicle for undoing language-induced confusions, cognition being of lesser relevance. But their analyses, if valid, could well be couched in a cognitive framework. Thus the debate on the status of logic in linguistic communication and reasoning is real and it persists to this day in philosophy, psychology, and linguistics (Montague 1974; Horn 1989; Monti and Osherson 2012).

George Lakoff described the issue succinctly: “Natural logic,” he said, “taken together with linguistics, is the empirical study of the nature of human language and human reasoning. It can have right and wrong answers” (Lakoff 1970). We took this dictum seriously and searched for relevant evidence from neuroscience.

We were also aware that the relation between linguistic and arithmetical abilities has similarly generated controversy (Chomsky 1988; Henschen 1920; Makuuchi et al. 2012; Changeux et al. 1998). To a cognitive neuroscientist, then, the question of whether logical, linguistic, and arithmetical abilities emanate from one and the same neurocognitive system is empirical, one that has not yet been fully resolved. These observations led us to an empirical excursion involving logic, language, and arithmetic that consisted of three stages: (I) functional: we produced behavioral and neuroimaging evidence pointing to the distinctness of linguistic, logical, and numerical functions in a group of healthy subjects (Dehaene et al. 2003; Deschamps et al. 2015). (II) Anatomical: we uncovered the neuroanatomical properties of a relevant cortical area in a series of postmortem brains, analyzed its microstructure, and created a three-dimensional, cytoarchitectonic map. (III) Integration of neuroimaging and microstructural data: we demonstrated a high degree of overlap between the anatomical and the functional, suggesting a unit of functional anatomy.

The functional distinctness of language and logic was investigated through sentences with and without logical negation, an elemental operation known to incur substantial processing cost (Deschamps et al. 2015). The task we used, moreover, enabled us to investigate the distinctness of numerosity from logic and language: participants heard sentences that required a comparison between two quantities and were asked to verify these sentences against visual images. To study the functional distinctness of arithmetic, we manipulated the difficulty of numerical comparison by tinkering with the properties of these visual images. This technique enables us to probe the brain’s numerical comparison modules and at the same time language and logic were probed. In sum, a single complex experiment allowed us to compare neural aspects of language, logic, and numerosity.

Indices for processing complexity and anatomical loci of activity were reaction time (RT) and fMRI signal intensity. Anatomical distinctness was studied by subjecting the area in the vicinity of the single region that the fMRI study uncovered (the left anterior insula) to cytoarchitectonic analysis and the *JuBrain* atlas (Amunts and Zilles 2015). This analysis revealed a new, cytoarchitectonically uniform brain area, which turned out to be distinct from regions known to support language and numerosity, but overlapped with the functionally defined negation area.

## Materials and methods

**Fig. 1:**
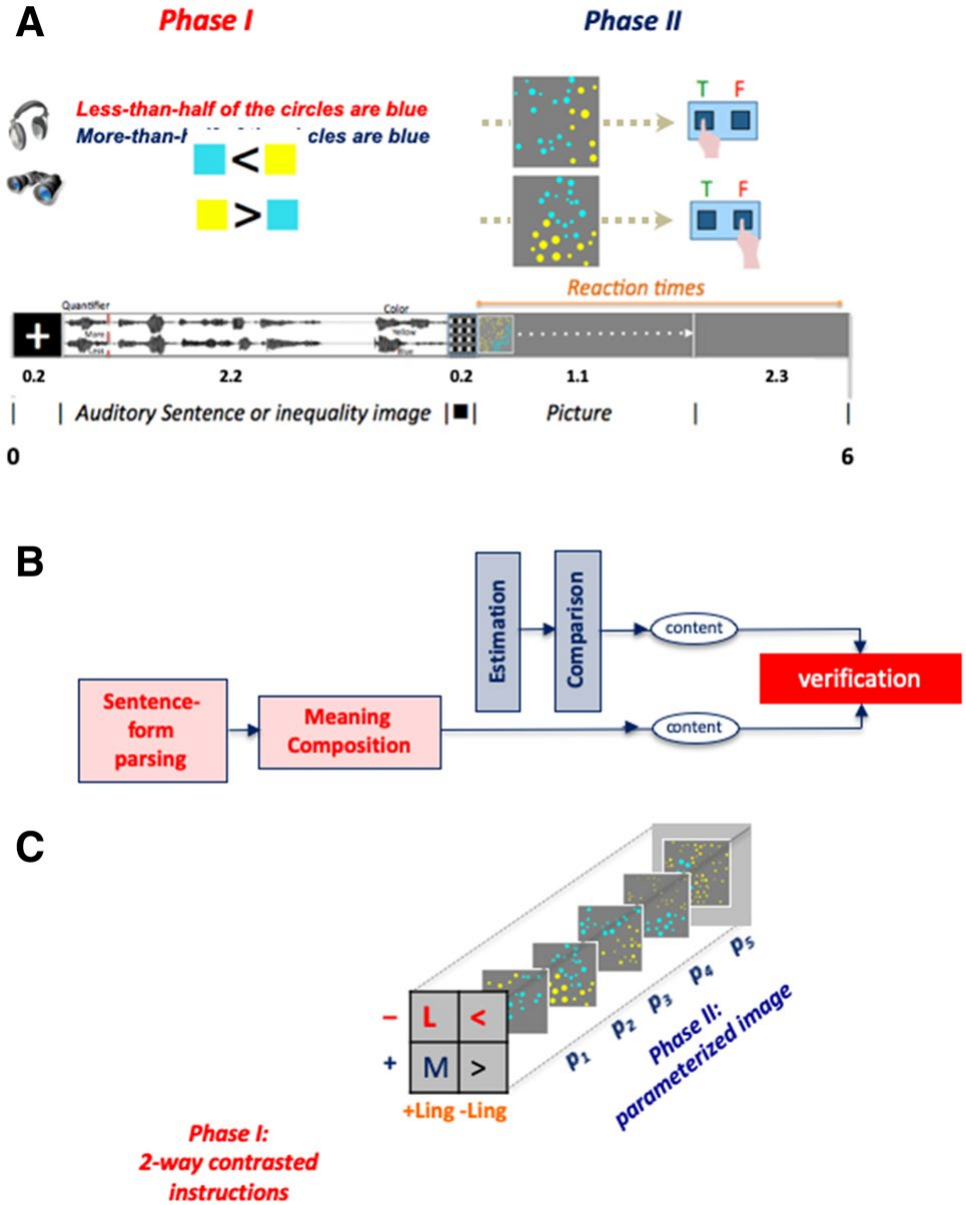
Design. **a** Trials: auditory sentence probes or incrementally presented visual expressions. At a fixed point, a proportion-depicting image appeared. Task: to indicate whether or not the image matched the probe. Auditory and visual probes had the same content. **b** The processes involved in the analysis of linguistic stimuli: Phase I triggered phonetic, morphophonological and syntactic analyses, followed by semantic analysis that produced content—a formal meaning representation encoding a proportion between two numerosities; Phase II required image analysis: estimation of the numerosities of the two clusters of colored circled, followed by a comparison. Verification was the step where participants decided about the match between Phase I and Phase II representations. **c** Design: the experiment featured three factors (Polarity, Probe type, Proportion). The first two had two levels each (**a**), each presented with six tokens of five different proportions, p_1_–p_5_

The fMRI experiment was approved by the ethics committee of the Montreal Neurological Institute (MNI). Twenty-three participants, recruited at McGill University, were tested. Two were excluded due to technical problems during scanning. All were right-handed as assessed by the Edinburgh Handedness Inventory (Russell 1903), native English speakers with normal hearing, and corrected to normal vision (mean age 23.6 SD = ± 4.5; range 19–35; 13 females). Participants gave informed consent in accordance with the ethics committee of the Montreal Neurological Institute (MNI) and the Helsinki Declaration of 1975 (revised 1983). The behavioral task performed inside the magnet is described in the text and in Fig. 1. The anatomical data were obtained from ten human postmortem brains (five males, five females). Donors, who had no clinical history of neurological or psychiatric disease, were reached through the body donor program of the University of Duesseldorf, in accordance to the guidelines of the ethics committee. A detailed description of the properties of the experimental materials, the behavioral, anatomical, and imaging methods and data analyses are given in the SI Appendix.

### Logical negation and language

Negation (¬) was defined by ancient Greek logicians as a logical operator that reverses the truth value of a proposition: if *p*, the proposition expressed by a sentence, is true, then ¬*p* is false and vice versa (Ross and Smith 1928). As a consequence, negation reverses the direction of inferences: *p *→ *q* is equivalent to *¬q *→ *¬p*. This can be demonstrated through a language example: Take *p *= *I own a dog*, *q *= *I own an animal*. If we assume that all dogs are animals ({*x*: *x* is a dog} ⊂ {*x*: *x* is an animal}), then *q* is true whenever *p* is true. We can therefore conclude that *p *→ *q*. But note that negation reverses the direction of the inference: *I don’t own an animal *→* I don’t own a dog*. Linguistic negation, then, produces effects in keeping with propositional logic, and specifically, abides by the well-known equivalence *p *→ *q*⇔*¬q *→ *¬p*. In cognitive neuroscience, studies have investigated the neural reflections of words that express negation (e.g., *no*, *not*) (Bahlmann et al. 2011; Tettamanti et al. 2008). Here, however, we sought evidence for a sharper distinction—we sought to dissociate language from logic. For that, we needed to *distill* logic from language—to extract logical operators from stimuli that contain them implicitly, even though they lack a corresponding word. Language avails us of tools to express negation implicitly. One of these is a class of polar quantifiers—quantity-denoting expressions that come in pairs, e.g., <*more*, *less*>. Here, we show that *less*, but not *more*, contains an *implicit* negation that is present logically (e.g., it reverses truth-value and thus inference direction), but absent morphophonologically (“Materials and methods”).

To be convinced that the meaning of *less* indeed contains implicit negation, note that if *he drank more beer than wine* is true, then *he drank less beer than wine* is false—truth value is reversed upon replacement of *more* by *less*. But this is not sufficient evidence. To be convinced that the meaning of *less* actually contains an implicit negation, let us use the inference reversal test demonstrated above for explicit negation (*not*): like in the affirmative sentence above, and given that all dogs are animals ({*x*: *x* is a dog} ⊂ {*x*: *x* is an animal}), we can safely infer from “*more than half of my friends own a dog*” that “*more than half of my friends own an animal*”. Now, consider how *less* induces an inference reversal, when it replaces *more*: if *less than half of my friends do not own an animal*, we can safely infer that *less than half of my friends do not own a dog*. This pattern of inferential relations between sentences, identical to those observed above with explicit negation, indicates that one member of the *more/less* pair implicitly contains a negation. Evidence adduced by semanticists indicates that *less* is the one (see “Materials and methods”). Seeking to test the separation between logic and language through negation, we constructed sentence stimuli that contained the complex quantifiers *more/less than half*.

Phase I sentence probes with *more* or *less*, thus had the same surface syntax, an identical number of words and syllables, and they unfolded in time in exactly the same way. The resulting meanings were identical up to negation, as their truth conditions were reversed—when one was true, the other was false and vice versa. Purely logical negation—which seems to be hidden in *less* (“Materials and methods”)—could thus be extracted. Our goal was to detect processing differences between *more* and *less*, and uncover the cerebral loci supporting these computations.

Yet, additional differences between the sentence probes required controls: *more* and *less* are different words with different lexical frequencies; as well, they induce reversed linear sequences of the two compared set cardinalities. These differences might matter. To control these differences, a pair of inequality symbols was used {<, >}, to construct non-language, quasi-algebraic, visual probes (Deschamps et al. 2015) that unfolded in time during Phase I (Fig. 1a). Probes with these elemental symbols were proper controls: like the quantifier pair, they denote a relation between two set cardinalities in reverse orders (*p *> *q *= *q *< *p*); unlike quantifiers, they are atomic, none contains a negation, and they have the same perceptual contour (“Materials and methods”).

Phase I probes thus featured a Probe type factor (+linguistic {*more, less*}; –linguistic {>, <}) and a Polarity factor (positive {*more,* >}; negative {*less,* <}). Our design dictated a focus on the processing signature of negation: the effect of negation (*less*–*more*) minus the specific symbol and reversal effects of the control conditions (<–>). That is, we expected to find a difference between differences or a Net Negation Interaction signature during Phase I—a Probe type *X* Polarity interaction between the two Phase I factors (*NetNegInt *= ∆_*“Less”*–*“More”*_> ∆_*“*<*”*–*“*>*”*_, which we will later cash in with specific units), which moreover stems from a negative Polarity signal that is higher than the positive.

### Verification and numerosity

Each trial in our bi-phasic speeded verification task had a Phase I auditory sentence or a visual quasi-algebraic expression that was about a proportion between two sets of colored circles. Phase II featured an image, depicting a proportion between quantities of blue and yellow circles (Fig. 1a). Participants were asked to verify the sentence against the image as fast as they could. This task required the comprehension of a probe in Phase I, numerical estimation of each of the two quantities in the Phase II image (blue/yellow circles) followed by a comparison between them, and a decision (Fig. 1b).

In the Phase II images, the numerosity of one quantity was fixed across images and the other was modified parametrically (Fig. 1c). The resulting blue/yellow proportions influenced task difficulty, leading to a Comparison effect (*Comp*): task difficulty (measured by the RT) was expected to predict the relative values of the dependent variables, as well as test whether these variables behave in accordance with Weber’s Law (Vogel et al. 2013). The two effects—*NetNegInt* and *Comp*—are already known to be independent (Deschamps et al. 2015).

The Phase I probes and the Phase II images had identical conceptual content and the proportion between blue and yellow circles made the probes true or false. Each part contributed its share to the overall processing cost, which was behaviorally indexed by RT, time-locked to the end of Phase I.

## Results

### Behavioral tests

**Fig. 2:**
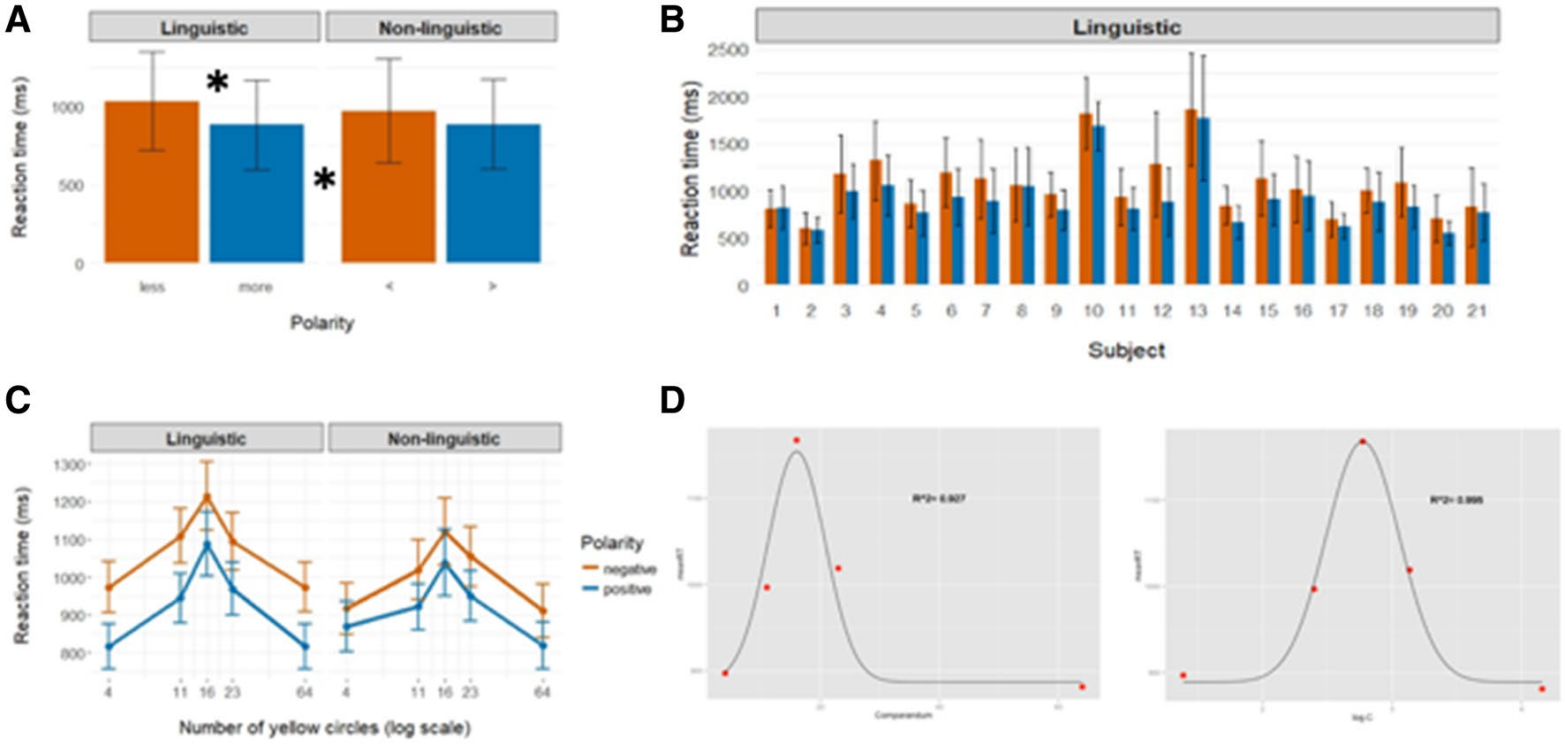
Behavioral results. **a** Group RT results by condition, across all image types (error bars: SEM). **b** The linguistic Polarity effect (RT_*less*_–RT_*more*_ across all blue/yellow proportions in the images) is very robust and is observed for most participants at the individual RT level (*n* = 21). Error bars mark SD. **c** Same group results, broken by proportion of circles visual probe (SEM). The x-axis is compressed logarithmically. **d** Gaussians fitted to the data from **c** before and after log compression show a greater *R*^2^ subsequent to compression, indicating compliance with Weber’s Law

The 21 participants who performed the verification task during the scanning session (“Materials and methods”), exhibited low error rates (84% ± 4% correct responses per participant across all conditions) and exhibited the *NetNegInt* signature (Fig. 2a): the negation effect within the linguistic pair of condition was very robust: mean RT_*less*_= 1035.3 ms (SD = 313.2); mean RT_*more*_= 882.8 (SD = 287) led to a very high significance on a paired-sample *t* test (*t*_(20_) = 7.33, *p* = 2.19e−0). The effect for the non-linguistic pair was less strong: mean RT_<_ = 971.4 ms (SD = 330.7); mean RT_>_ = 888 ms (SD = 277.7), a paired-sample *t* test (*t*_(20)_ = 3.947, *p* = 0.0008). The linguistic effect, moreover, was manifested individually for almost all participants (Fig. 2b), as well as across all blue/yellow proportions in the Phase II images (Fig. 2c). The judgment times (on a logarithmic scale) were analyzed in a repeated-measures ANOVA. The *NetNegInt* signature thus took the following shape, replicating previous results: (1) a significant Probe type *X* Polarity interaction (*F*(1,20) = 9.18, *p* = 0.007); (2) the source of the interaction was: ∆RT_*“Less”*–*“More”*_> ∆RT_*“*<*”*–*“*>*”.*_

An additional factor that affected RT was the proportion between the number of blue and yellow circles in the image: the experiment featured five *red/yellow* proportions (p_1_–p_5_ in Fig. 1c), sentence verification required image scanning and numerical comparison, which is subject to this law (Fig. 2d). Thus, we could test whether participants performed in keeping with Weber’s Law. Mean RT across participants for the verification of each proportion was computed, a Gaussian was fitted to these means and the coefficient of determination (*R*^2^) was calculated. The fit of the performance curve to a Gaussian improved when the *x*-axis (blue/yellow proportion) was logarithmic (*R*_log compressed_^2^ = 0.995, right panel) compared to linear (*R*_linear_^2^ = 0.927, left panel).

The effects of negation and proportion on RT were independent: a Gaussian with three parameters (baseline, amplitude and width) was fitted to the set of RT values of the *more* and *less* conditions (Fig. 2b) and a permutation test was conducted. The baseline parameter reflects RT effect due only to negation. Only this parameter yielded a significant difference between the conditions (*p* < 0.00003), indicating that RT effects due to proportion and those due to negation were independent. Past behavioral results (*NetNetInt* signature), as well as results pertaining to the modularity of linguistic and arithmetical processing, were thus replicated, this time during an imaging session (Deschamps et al. 2015).

### Fmri

The analysis of fMRI data had two goals: (1) to identify brain loci supporting logical negation, the *NegNetInt* signature as a proxy; (2) to test the neural separation between linguistic, logical, and numerical processes. Our design, in which probes and images were present sequentially (Phases I, II), enabled to distinguish them analytically.

#### Phase I

A whole brain analysis of the Phase I BOLD activity patterns revealed two clusters that manifested the *NetNegInt* signature:

- at the left anterior insula (*NetNegInt*: *F*_1,20_ = 21.84, *p* = 0.00015, corrected, see “Materials and methods”; Fig. 4b, Phase I panel). In this cluster, a main Polarity effect was also found (*F*_1,20_ = 38.04, *p* = 0.000005, corrected). In the linguistic conditions, the activation of the negative quantifier (PSC = 0.25%, SEM = 0.031) was higher than that of the positive one (PSC = 0.2%, SEM = 0.03). A paired-sample *t* test was highly significant (*t*_(20)_, *p* < 0.0000001). No significant difference between the non-linguistic conditions (<, >) was found.
- at the Superior Temporal Gyrus (STG), (*NetNegInt*: *F*_1,20_ = 37.44, *p* = 0.000006, corrected, Fig. 4c, Phase I panel). This cluster also exhibited a main effect of Polarity (*F*_1,20_ = 10.10, *p* = 0.005), where the negative quantifier produced a higher activation (0.21 ± 0.22) than the positive quantifier (0.13 ± 0.22), a statistically significant increase of 0.07 ± 0.05 (*t*_(20)_ = 6.3, *p* = 0.000004). No significant difference between the non-linguistic conditions <, > was found.

#### Phase II

**Fig. 3:**
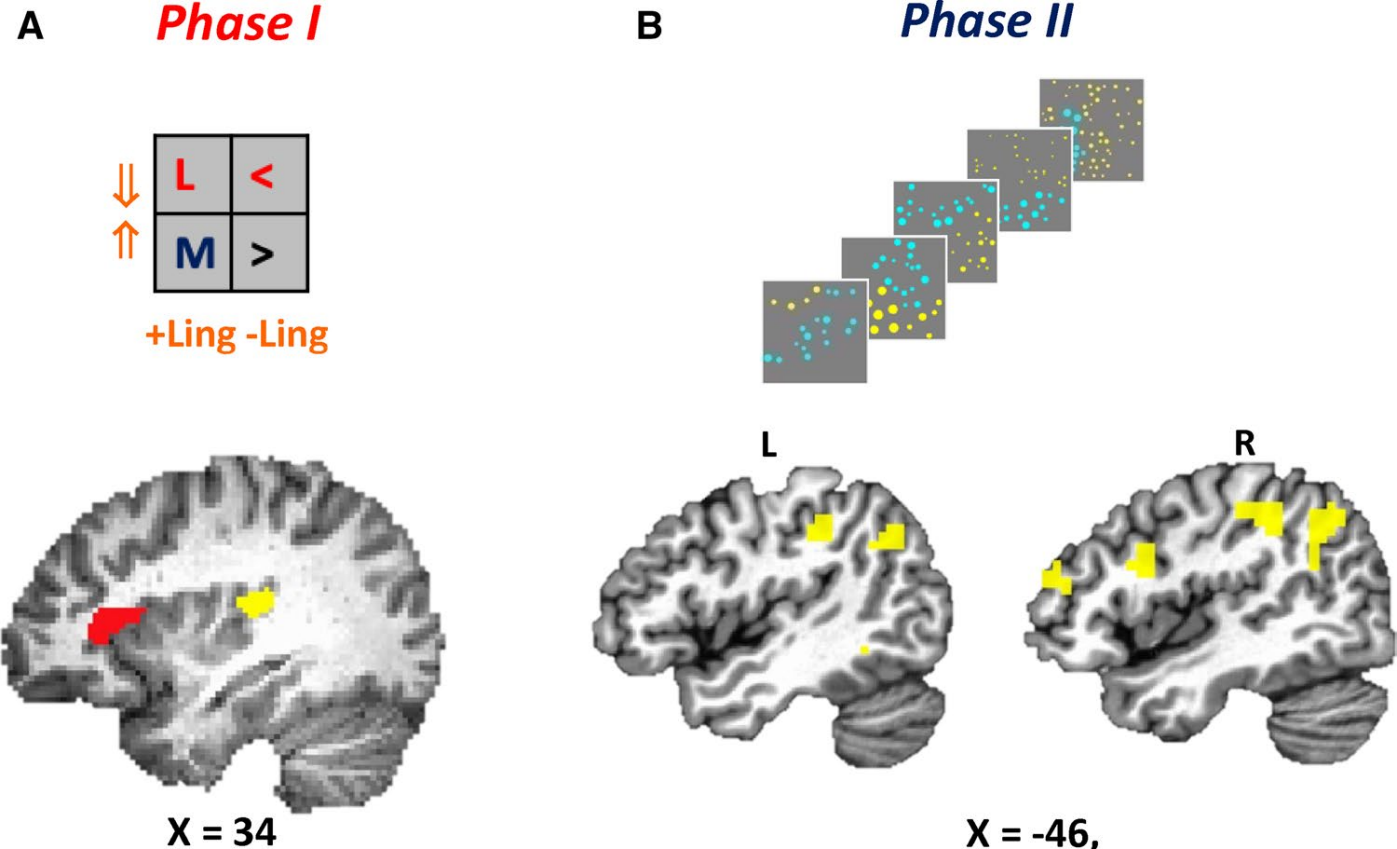
FMRI results, broken by phase. **a** Phase I *NetNegInt* clusters: (1) in the left anterior insula (red), where a main Polarity effect (*F*_1,20_ = 38.04, *p* = 0.000005) and a Polarity *X* Probe type interaction (*F*_1,20_ = 21.84, *p* = 0.00015); (2) in the left Superior Temporal gyrus (yellow), with a main effect of Instruction (*F*_1,20_ = 8.16, *p* = 0.010), a Phase I main effect of Polarity (*F*_1,20_ = 10.10, *p* = 0.005) and an interaction between Instruction and Polarity (*F*_1,20_ = 37.44, *p* = 0.000006). **b** Phase II *Comp* effect: the left and right clusters in which a *Comp* effect was significant (Table 1). A conjunction analysis between the *Comp* and *NetNegInt* clusters revealed no overlap

The Phase II BOLD response for numerical comparison (*Comp*), measured during and after image display, was detected using RT as a predictor (“Materials and methods”). As task difficulty was in keeping with Weber’s Law (Fig. 2d), RT could be used as a proxy for the *Comp* effect (Vogel et al. 2013). A parietal *Comp* effect was recorded bilaterally (Table 1, Fig. 3b). This result, while new for the present task, is consistent with previous ones that have identified similar regions as supporting numerical comparison (Heim et al. 2012; Piazza et al. 2004).

**Table 1:** Phase I and Phase II activation clusters

Phase	Location description	JuBrain Atlas region	p-JuBrain	Hemisphere	*X*	*V*	*i*	If voxels	*F*/*t*	*p*
1	Superior temporal gyrus and transverse temporal gyrus	AUDITORY_TE21 AUDITORY_TE22	0.421	Left	− 54	− 24	6	55	10.38	0.004
	Anterior insula	INSULA IAD1	0.911	Left	− 30	26	6	43	20.51	0.0002
II	Superior parietal lobule	IPS_JP3	0.13							
		SPL_7	0.367							
		SPL_7A	0.262							
		SPL_7PC	0.104	Right	34	− 58	58	177	3.17	0.004
	Angular gyrus			Left	− 36	− 76	52	95	− 4.81	0.0001
	Paracentral lobule			Right/left	2	18	52	93	4.68	
	Angular gyrus			Right	44	− 72	54	66	− 3.25	0.004
	Inferior frontal gyrus, pars operculars	BROCA_45_R	0.284							
		IFS_IFJ1	0.217	Right	50	12	34	45	3.67	0.002
	Supramarginal gyrus and intraparietal sulcus	IPS_JP2	0.485							
		IPL_PFT	0.267	Left	− 48	− 38	44	37	3.74	0.001
	Superior temporal gyrus and middle temporal gyrus	AUDITORY_TE3	0.200							
		BR0CA_44	0.127							
		OPERCULUM_OP4	0.263	Right	64	2	4	26	− 3 68	0.001
	Middle temporal gyrus and superior temporal gyrus									
				Left	− 68	− 48	− 4	26	− 4.05	0.0006
	Middle frontal gyrus			Right	50	40	20	26	3.52	0.002

Coordinates (MNI space) represent the maximum peak value for each functional cluster (minimum cluster size of 25 contiguous voxels each significant at *p* < 0.005). p-JuBrain represents the probabilistic value for the peak functional voxel to be within the JuBrain atlas region

**Fig. 4:**
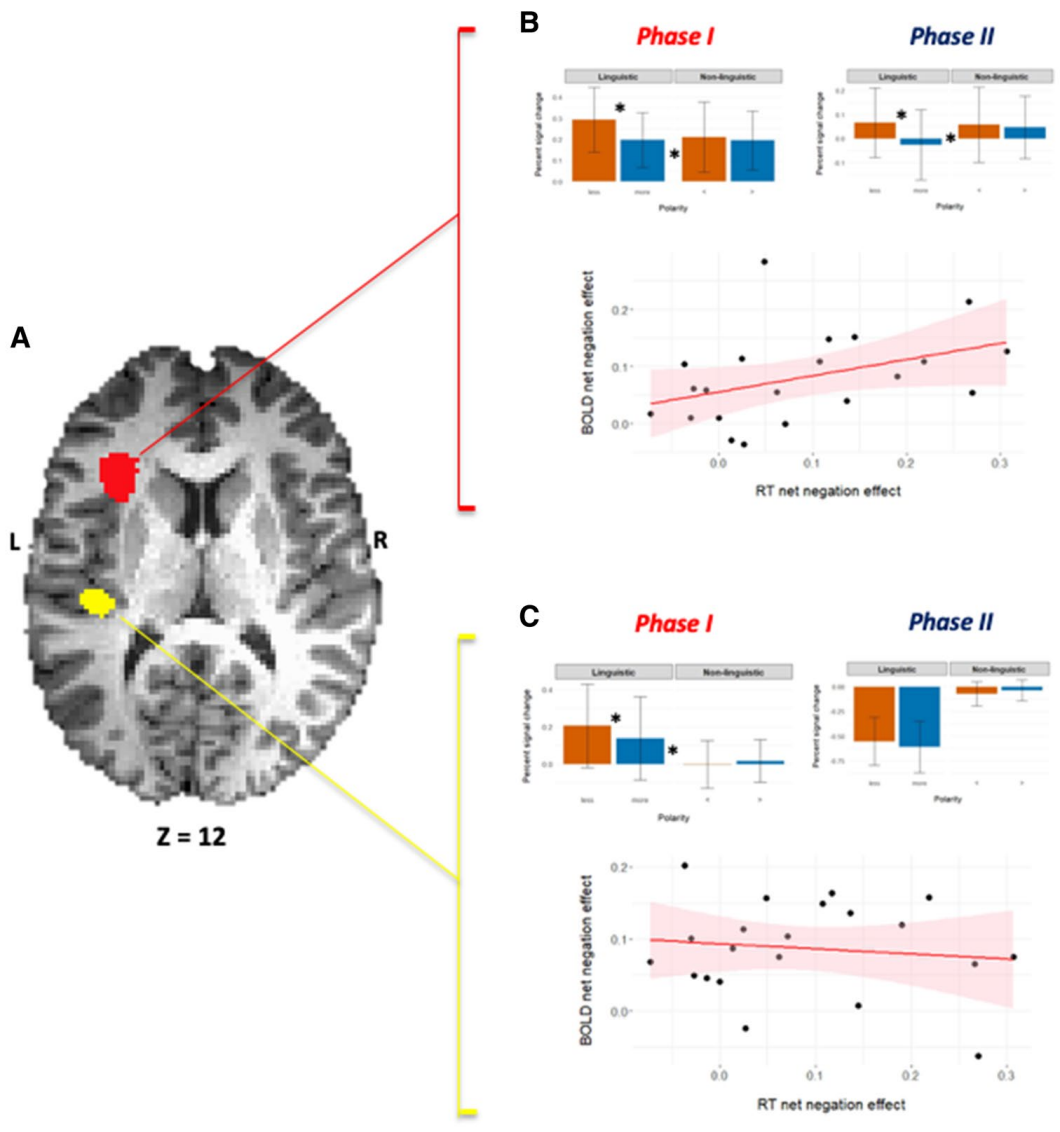
Comparing the modus operandi of two *NetNegInt* clusters. **a** An axial view of both clusters. **b** Phase I and Phase II imaging results for the *NetNegInt* cluster in the left anterior Insula (z = 12)—Group Percent Signal Change (PSC) by condition (error bars: SEM). A main Polarity effect (*F*_1,20_ = 38.04, *p* = 0.000005) and a Polarity *X* Probe type interaction (*F*_1,20_ = 21.84, *p* = 0.00015) were found (top), as well as a positive correlation between individual behavioral (RT) and fMRI (BOLD) *NetNegInt* effects. **c** Phase I imaging results for the *NetNegInt* cluster in the STG (top) and no correlation between behavioral and fMRI *NetNegInt* effects (*r* = − 0.12, *p* = 0.603)
- at the STG, all activations were negative. Such deactivations are typically associated with the Default Network (of which the STG is sometimes thought to be part) and their interpretation in the context of a task remains elusive. Importantly, though, this all-around negative pattern is substantially different from any of the activation patterns reported above.

Next, we explored the Phase II activation patterns of the two *NetNegInt* masks from Phase I (Fig. 3a) to see whether the *NetNegInt* signature persists across both phases. The cluster on the left anterior insula (*NetNegInt* cluster henceforth) was the only one to exhibit this signature, indicating that negation is still active in the representation to be verified at trial’s end:

- at the left anterior insula (*NetNegInt*: *F*_(1,20)_ = 31.003, *p* = 0.000019, corrected, Fig. 4b, Phase II panel). A paired-sample *t* test for the linguistic condition compared the percent signal change (PSC) for the negative quantifier highly significant (*t*_(20)_ = 8.698, *p* = 0.00000003, 2-tailed), with the negative condition producing higher activation than the positive one. No difference was found for the non-linguistic condition (<, >, *t*_(20)_ = 0.762, *p* − 0.455).

### Behavior/signal intensity correlations

In the insular *NegNetInt* cluster, the fine behavioral and fMRI *NetNegInt* effects, calculated at the individual participant level, were correlated in both phases and were found to be significant for both phases (Phase I: *r* = 0.402, *p* = 0.035, Fig. 4b bottom panel; Phase II: *r* = 0.432, *p* = 0.025, both 1-tailed). The same analysis, carried out on the STG cluster, detected no significant behavioral/fMRI signal intensity correlation (Phase I: *r* = − 0.12, *p* = 0.603, Fig. 4c, bottom panel; Phase II: *r* = − 0.193, *p* = 0.2).

### Uniqueness and cohesion of the insular cluster

In sum, the left anterior insular *NetNegInt* cluster was unique in exhibiting three critical properties that indicate functional cohesion: (a) the *NetNegInt* signature. (b) A significant positive correlation between the individual *NetNegInt* brain activity index (BOLD signal intensity) and its behavioral RT analog. (c) Persistence of both effects across Phases I–II, that is, during both the construction of a meaning representation and its verification against the image.

Next, the functional uniqueness of the insular *NetNegInt* cluster in both Phases I and II was tested, by identifying putative joint activities with clusters associated with other parts of the tasks. First, conjunction analyses on both the insular *NetNegInt* and *Comp* functional clusters detected no significant joint activity, indicating the anatomical disjointness of logical and numerical operations.

Finally, anatomical ROI analyses of *JuBrain*-defined areas 44, 45, viewed as classical language areas (Amunts et al. 1999; Amunts et al. 2004), were also conducted for both the *NetNegInt* and the *Comp* effects. No significant effect was found in the region of both anterior language areas, indicating the anatomical disjointness of logical and linguistic operations. Third, the left temporal pole, a region claimed to support combinatorial semantics (Anderson et al. 2017; Del Prato and Pylkkänen 2014), was silent, but began to surface as an active *NetNegInt* site once the threshold was dropped to a low, uncorrected *p* < 0.07. Linguistic activity, logical negation, and numerical operation (numerosity), then, were clearly dissociated. This uniqueness and cohesion pointed to the left anterior insula as a new region of interest, which we proceeded to characterize anatomically.

### Cytoarchitectonic map

**Fig. 5:**
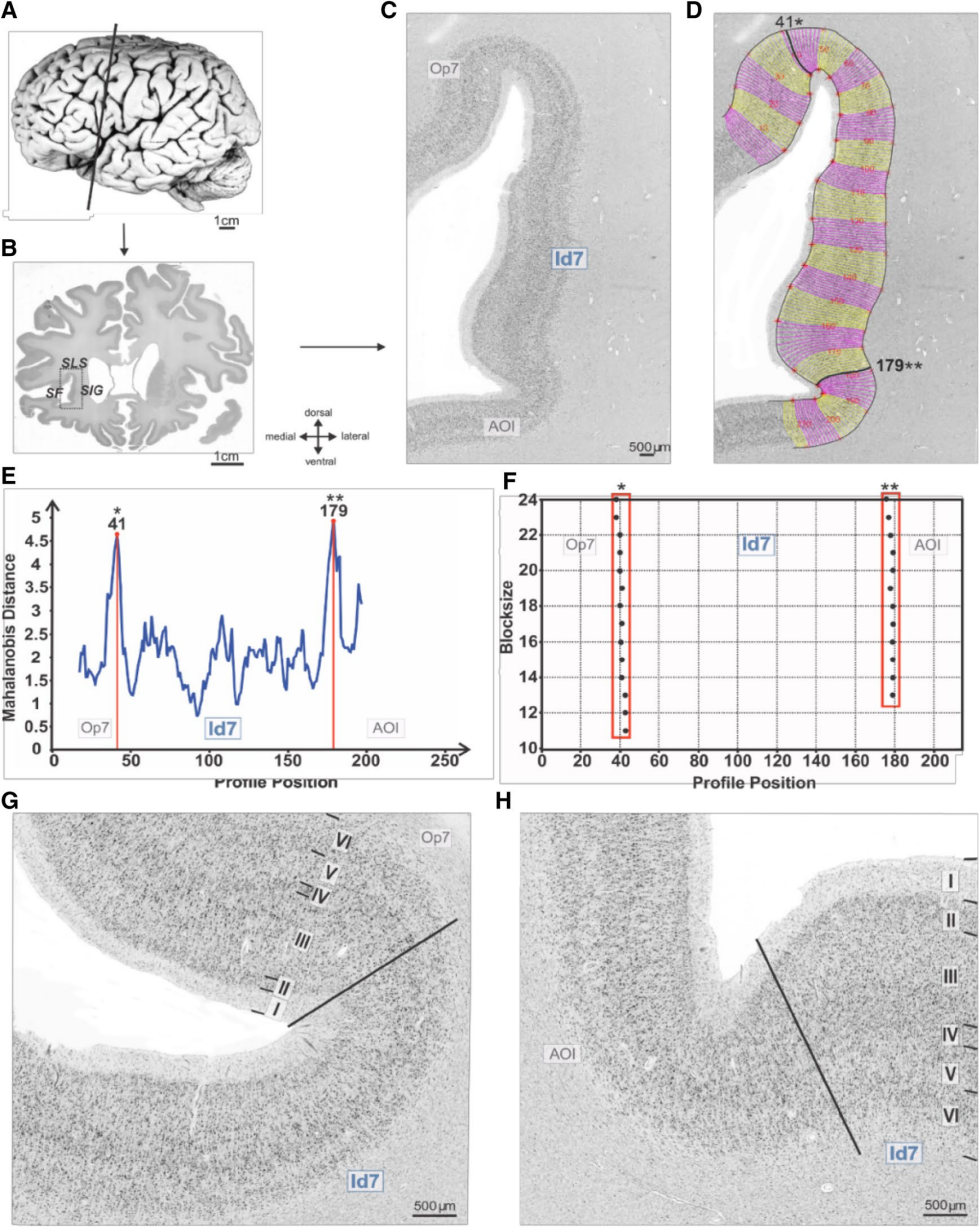
Image acquisition and algorithm-based definition of areal borders. **a** Left lateral view of a postmortem brain (*n* 2); position of histological section n 4891 is indicated. Scale bar: 1 cm. **b** Cell body-stained coronal section shown in **a**, the box indicates the region of interest (ROI), shown in **c**; scale bars: 1 cm and 500 µm, respectively. **d** GLI profiles cover the ROI from layer I/II to layer VI/white matter. Black lines localize the significant maxima of the Mahalanobis distance function, as quantified in **e**. **f** Position (*x*) of the significant maxima of the Mahalanobis distance plotted against blocksize (*y*). Vertical frames correspond to the accepted borders. **g** Cytoarchitectonic border between areas Id7 and Op7. The border is characterized by an increase in density of pyramidal cells in deep layer III and a higher packing of multiform cells in layer VI of Op7. **h** Cytoarchitectonic border between areas Id7 and AOI. This border is characterized by a decrease in neuronal density, in deeper layers III and V of AOI. Roman numerals indicate cortical layers. Scale bar: 500 µm. *SLS* Superior Limiting Sulcus, *SIG* Short Insular Gyrus, *SF* Sylvian Fissure, *Op7* opercular area 7, *Id7* insular dysgranular area 7, *AOI* area orbito-insularis

**Fig. 6:**
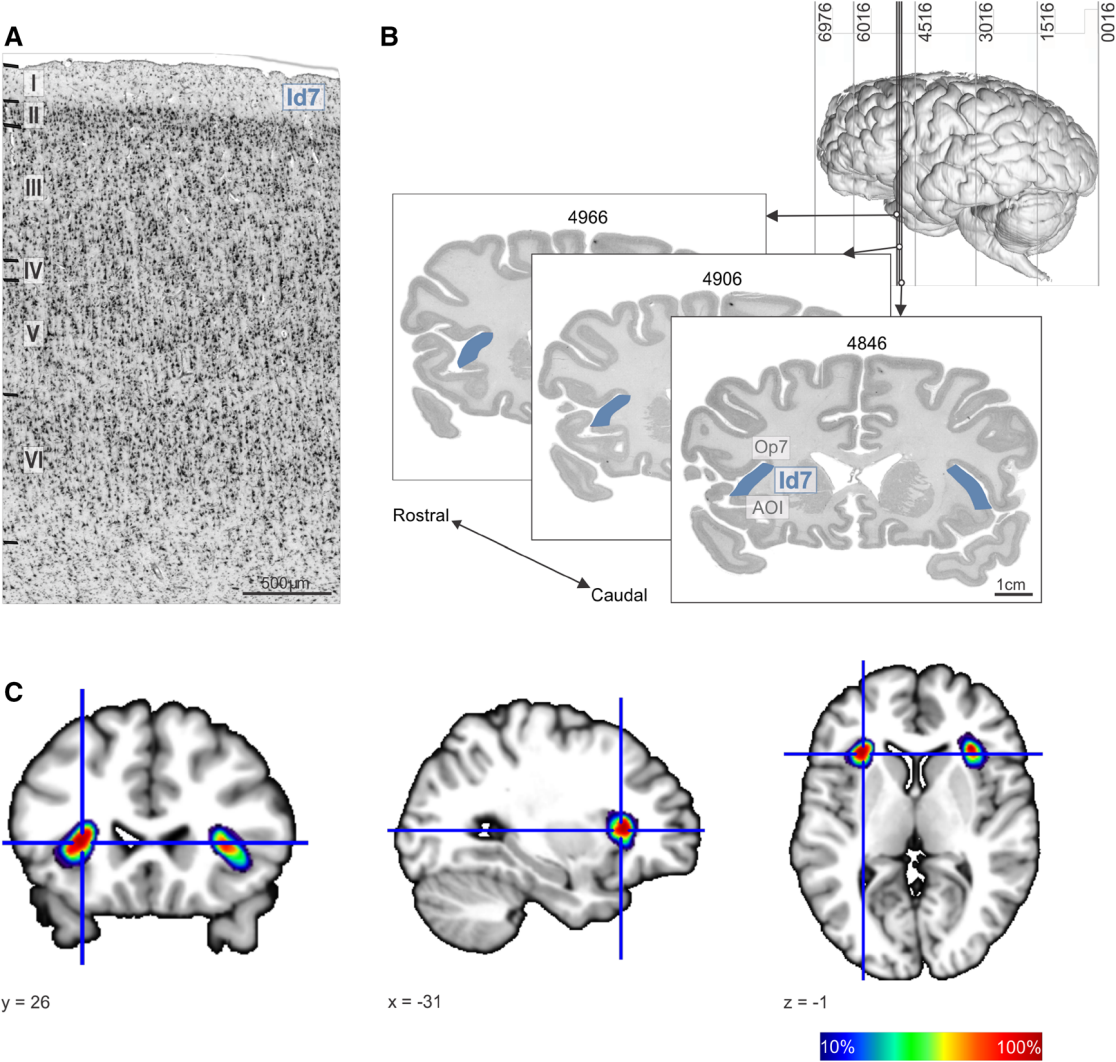
Cytoarchitecture, probability maps of Id7 and location of the anatomical cluster. **a** The dysgranular area Id7 presents a discontinuous layer IV, interrupted by pyramidal cells from layers III and V. In the latter layers, two distinct sublayers can be distinguished. Roman numerals indicate cortical layers. Scale bar: 500 µm. **b** Caudal-rostral sequence of three coronal sections, from brain *n* 12 (Table S3), displaying the extent of area Id7 (in blue); on top right, a lateral view of the 3D reconstructed brain. Scale bar: 1 cm. **c** Probabilistic maps of Id7 in representative coronal, axial, and sagittal sections. Dark red and dark blue regions represent, respectively, areas with high (9–10 brains) and low overlap. The coordinates correspond to the stereotaxic position of the sections in anatomical MNI space (63)

To identify the precise anatomical correlates of this region, we analyzed cytoarchitectonically the anterior insula in histological sections of ten human postmortem brains (five males, five females; Table 2). Image analysis and an observer-independent procedure relying on multivariate statistical analysis were used to define areal borders and quantify inter-areal differences (Amunts et al. 1999). We identified and mapped a new cytoarchitectonic area, Id7 (Insular dysgranular area 7), located on the latero-dorsal surface of the anterior short insular gyrus of both hemispheres (Figs. 5, 6). Area Id7 is a six-layered, dysgranular area, characterized by an interrupted and inconspicuous inner granular layer (layer IV). The mean volume of Id7 is 421 mm^3^ (SD = 146) on the left and 354 mm^3^ (SD = 129) on right hemisphere (corrected for shrinkage). Inter-hemispheric cytoarchitectonic analysis did not show any significant left–right asymmetry (paired sample *t* test = *p *> 0.05; “Materials and methods”).

**Table 2:** Postmortem brains used for the cytoarchitectonic analysis of area Id7

Case no.	Age in years	Gender	Cause of death	Brain weight (before fixation)	Fixative
4	75	M	Toxic glomerulonephritis	1349	Formalin
5	59	F	Cardiorespiratory insufficiency	1142	Formalin
6	54	M	Myocardial infarction	1757	Formalin
7	37	M	Right heart failure	1437	Formalin
8	72	F	Kidney failure	1216	Formalin
9	79	F	Cardiorespiratory insufficiency	1110	Bodian
10	85	F	Mesenteric artery infarction	1046	Bodian
11	74	M	Myocardial infarction	1381	Formalin
12	43	F	Pulmonary embolism	1198	Formalin
13	39	M	Drowning	1234	Formalin

### Precise functional anatomy of negation

**Fig. 7:**
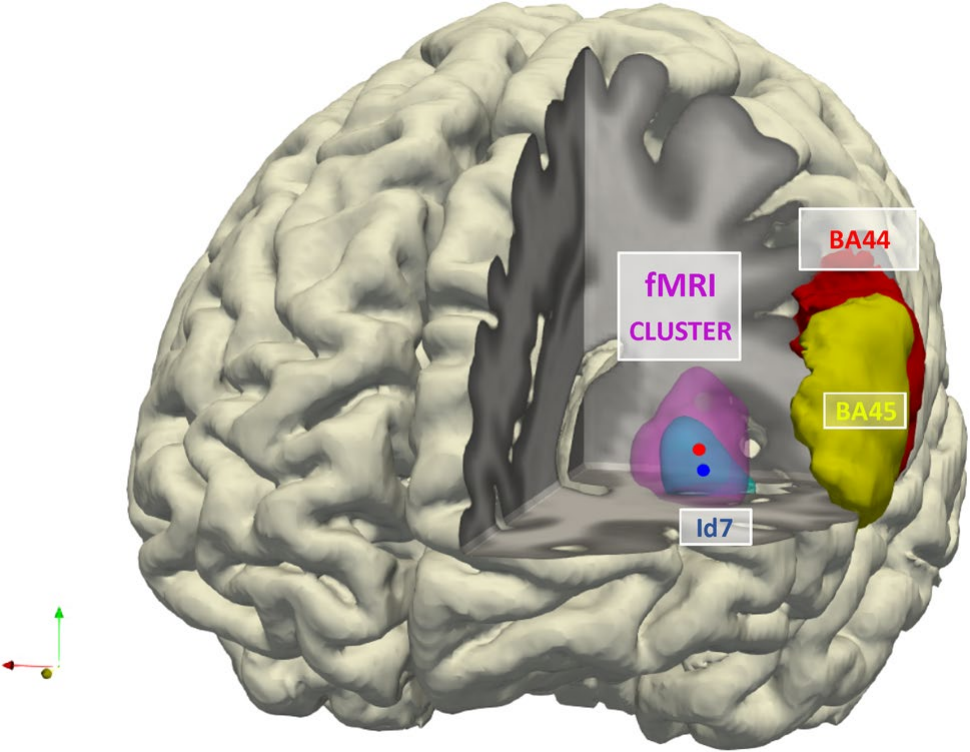
Anatomical region and Phase I functional activation in our task. An expansion of Fig. 3d: the visualization here is a three-dimensional reconstruction of a brain: the left frontal lobe is gradually removed to show the spatial relations between the fMRI activation cluster (magenta) and the cytoanatomically defined region Id7 (blue), as well as areas 44 (red) and 45 (yellow) in Broca’s region. The blue and red dots indicate the centers of mass of region Id7 and the fMRI cluster, respectively. The functional activation cluster and area Id7 overlap to a large extent, and are clearly separated from areas in Broca’s region. *Op7* opercular area 7, *Id7* insular dysgranular area 7, *AOI* area orbito-insularis, *BA44* Brodmann’s Area 44, *BA45* Brodmann’s Area 45

Next, the functional cluster was superimposed on the new anatomical area, to study the extent of overlap and topographical relationship. The voxels contained in both *NetNegInt* cluster and Id7 amounted to 29.2% of the anatomical volume and to 19.2% of the functional volume (once resampled for 1 mm^3^ anatomical voxels). The remainder, being in the white matter, was deemed to be a methodical artifact. Both clusters excluded all other cortical regions, in particular, areas 44 and 45 of Broca’s region (Fig. 7). The *NetNegInt* cluster’s peak of activation (− 30, 26, 8) was located within the anatomical Id7 at a probability *p* = 0.91, substantially higher than any analogous value found (Table 1). Moreover, the Centers of Mass of the anatomical probabilistic map (− 32, 23, 4) and the *NetNegInt* functional cluster (− 32, 26, 8) were closely related to one other (Fig. 7, see Supplementary Movie).

Finally, an anatomical ROI-based analysis, using a threshold map of the left Id7 (“Materials and methods”), also revealed a significant *NetNegInt* effect (*F* = 6.258, *p* < 0.02).

## Discussion

Taken together, the anatomical and functional clusters exhibit bi-uniqueness: area Id7 is cytoarchitectonically distinct from its neighbors, and represents a new, independent cortical area of the anterior insula (Fig. 7, Movie). The functional *NetNegInt* coincides largely with Id7, overlaps with no other cortical region, and *NetNegInt* intensity correlates with RT at the individual participant level.

At a minimum, these results allow us to conclude that there is a single, anatomically and functionally cohesive core area involved in negation—Id7/*NetNegInt*. It is distinct from areas 44, 45, long believed to support syntax and from areas supporting core compositional semantic processes in the left temporal pole (Del Prato and Pylkkänen 2014). This distinctness and cohesiveness illustrates how relatively small elements of cognition can be neurally individuated and correlated with cytoarchitectonically defined areas. It also supports a modular view of cognitive functioning (Fodor 1983) and moreover seems to provide an answer, albeit partial, to the perennial debate about language and logic. If evidence from neuroscience bears on the debate, then Frege, Russell, and their followers were right: language and at least some aspects of logic are distinct. Finally, our results suggest that the border between the insula and Broca’s region is where language stops and logic begins.

We are not in a position to establish a connection between our results and other roles attributed to the anterior insula such as interoception. Yet, there is a difference in pattern: typically, the anterior insula is activated bilaterally (Zaccarella and Friederici 2015), and tends to co-activate with the anterior cingulate (Craig 2009; Engstrom et al. 2014), to which the left and right insulae appear to be massively connected (Mesulam and Mufson 1982) and have a similar histologic makeup (Ghaziri et al. 2017). Our study documented no bi-lateral co-activation. Recent lesion data, moreover, relate interoceptive deficits to regions that seem to exclude the here defined left Id7 (Salomon et al. 2018).

So what can we conclude and where do we go from here? Our experiment demonstrates that the processing of one logical connective, ¬, has a distinct neurocognitive signature, supported by a histologically coherent piece of neural tissue, the left Id7, that is, outside the traditional language regions, lying between them and decision making areas. While we believe that this set of findings provides the basis for an important argument for language–logic dissociation, we are aware that it is based on a single set of results, one that needs to be further enriched in the same spirit. Convergent results from related explorations of other logical connectives will no doubt help to bolster our claims. E.g., if experiments can be designed to successfully isolate disjunction, conjunction, and the like, and their results converge, solid foundations for a new perspective on language–logic relations would be constructed.

With this qualification, can we conclude that the philosophers were right? Gottlob Frege, in his *Begriffschrift*, famously asserted that linguistic rules relate to logic as the eye compares to a microscope (van Heijenoort 1967): language is flexible, but logic is more rigid—mediating between linguistic expressions and objects suitable to reasoning. While Frege and Russell had no cognitive perspective, let alone a neurological one, we feel free to add one and assign an anatomical construal of Frege’s assertion in regards to the spatial position of the left Id7: like a microscope, this area may “translate” linguistic objects into logical forms. A mediating role has already been proposed for the posteriorly adjacent, middle left insula, claimed to mediate between motor planning and speech (Dronkers 1996). In a similar vein, it is proposed that the left Id7 mediates between the language regions and prefrontal areas engaged in reasoning (Baggio et al. 2016; Monti et al. 2007). By doing so, it seems to play a crucial role in what could be a core neural network that underlies our humanity.

## Electronic supplementary material

Below is the link to the electronic supplementary material.

Supplementary material 1 (DOCX 333 kb)

Anatomical region and functional activation in logical processing task: video expansion of Fig. 7 (MOV 6708 kb)

## Data Availability

Analysis scripts, to reproduce the reported results, are available through the first author’s account (https://www.grodzinskylab.com/data-and-code). The corresponding datasets are available on reasonable request. The cytoarchitectonic probabilistic map is available to the research community, e.g., through the JuBrain atlas and the HBP Human Brain Atlas (https://www.humanbrainproject.eu/en/explore-the-brain/atlases/).
