# Supplementary material for: Logical negation mapped onto the brain
Source: Brain Struct Funct. 2019 Nov 4;225(1):19–31. doi: 10.1007/s00429-019-01975-w (PMC6957563; doi:10.1007/s00429-019-01975-w)
Supplement: Supplementary file 1 — Supplementary material 1 (DOCX 333 kb) [file 429_2019_1975_MOESM1_ESM.docx]

Supplementary Information for

**Logical negation mapped onto the brain**

Authors: Yosef Grodzinsky, Isabelle Deschamps, Peter Pieperhoff, Francesca Iannilli, Galit Agmon, Yonatan Loewenstein, Katrin Amunts

Corresponding author: Yosef Grodzinsky

Email: [yosef.grodzinsky@mail.huji.ac.il](mailto:yosef.grodzinsky@mail.huji.ac.il)

**This part of our submission includes:**

Supplementary text

Table S1

Captions for Movie S1

References for SI reference citations

**Other supplementary materials for this manuscript include the following:**

Movie S1

Supplementary Information Text

**Trial design**

Trials were made up of 2 phases: Phase I contained an instruction probe, consisting of either an auditory sentence or a written quasi-algebraic inequality. Phase II contained an image with blue circles and yellow circles of varying numerosities (Figure 1, main text). Speeded verification required an estimation of these two numerosities, and a comparison between them. The sequential presentation enabled the neural separation of concurrent mental operations at the level of complex combinations; it also allowed to track the neural dynamics of verification.

**Phase I instruction probes**

*Sentences and quasi-algebraic inequalities*

Four pairs of expressions were verified against visual scenarios, with two Phase I factors (Polarity and Probe Type) (Methods Table 1):

| ***Probe Type*** | | | |
| --- | --- | --- | --- |
| ***Polarity*** |  | *+Linguistic* | *–Linguistic* |
|  | + | a. More-than-half of the circles are blue/yellow | c. ■>■; ■>■ |
|  | – | b. Less-than-half of the circles are blue/yellow | d. ■<■; ■<■ |

**Table S1. Factors and stimulus types.** Each of the 4 different probes was presented with yellow and with blue as target color, yielding a total of 8 different probes (2 quantifiers × 2 colors + 2 inequalities × 2 colors).

*The language probes*

Negation reverses truth value: if *p* is true, then *not(p)* is false. It also reverses the direction of inferences (*p*→*q* ⇔ ¬*q*→¬*p*):

(1) a. p is TRUE iff ¬p is FALSE (p=1 iff =0)

b. *p*→*q* iff *¬q*→*¬p*

This reversal function is typically mediated by a negation word, therefore, a word distinguishes an affirmative sentence from its negated counterpart. This must be controlled, and at the same time, the 2 sentences must be identical up to negation, hence they must be semantically equivalent. Sentences *p* and *q* are semantically equivalent if they entail each other. That is, whenever *p* is true, *q* is true and vice versa.

Natural language sentences demonstrate identical behavior. For example, the set of boys who are both students and runners is a subset of the set of boys who are students {x/x=boy & x=student & x=runner}⊆ {x/x=boy & x=student}. This is intuitively clear, e.g., there may be boys who are students but not runners. Thus, the inference in the negation-less (2a) in valid, taking us from a subset (student runners) to a superset (students). Importantly, in the negation-containing (2b), the direction of the inference is *reversed* – from students to student runners:

(2) a. **Every** boy in the room was a student and a runner

⇒ **Ever**y boy in the room was a student

b. **Every** boy in the room was **not** a student

⇒ **Ever**y boy in the room was **not** a student and a runner

The same behavior – reversal of the direction of the inference – persists when “**every**” and “**every**…**not**” are replaced with “**more**” and “**less**”, respectively. While a visible (or audible) negation is absent, the inference reversal is evidence for its abstract existence:

(3) a. **More-than-half** of the boys were students and runners

⇒ **More-than-half** of the boys were students

b. **Less-than-half** of the boys were students

⇒ **Less-than-half** of the boys were students and runners

An abstract negation is therefore considered to be part of **Less** ^1,2^, which sets it apart from **More**, its control in our study. Both were used to build proportional quantifiers, a move that enabled the construction of meaning equivalences. In a universe of discourse that contains blue and yellow circles, and nothing else, sentences (4a-b) are equivalent, have an identical number of words and syllables, and yet, (4b) contains a negation that (4a) does not:

(4) a. More-than-half of the circles are blue

b. Less-than-half of the circles are yellow

Negation-containing expressions incur greater processing cost than their non-negated counterparts, has been shown, first by Just & Carpenter^3^, and then in further detail and precision by Deschamps et al.^4^, who demonstrated that covert negation has a measurable, robust processing cost, that is independent of the properties of the scenario that makes it true or false.

*The non-language probes*

An inequality is a relation holding between two values, or quantities, if they are different. In this experiment, the symbols *<*, *>* were used for reversal control. These symbols are used to compare the size of the values that flank them, and mark the direction of the difference. We used them because their denotation is said to be the same as *less* and *more* respectively, and because each is the converse of each other. Of interest to us is the fact that the direction of an inequality remains unchanged if we multiply by the identity element, multiplication by its negation reverses the inequality (5):

(5) a. if a>b, then 1*a>1*b

b. if a<b, then –1*b<–1*a

Importantly, *<*, *>*, whose geometrical contours are identical, share no other asymmetry that holds between *less* and *more*. Importantly, there is no way to decompose any of them into the other plus (or minus) negation, which is why this pair of inequalities is a proper control for our experiment.

**Phase II images**

*Numerical properties of the images*

For each visual scenario, the value of the *reference* numerosity *r* (the one mentioned in the sentence) was fixed at 16, while the value of the other, *comparandum* numerosity *c* (the other color in the image) varied parametrically around *r*=16 (that is: *c*= 4, 11, 16, 23, and 64), resulting with 5 *r/c* proportions (p_1_- p_5_ in Figure 1, main text). A total of 24 visual displays were created for each ratio, using a Mathematica™ script, half of which were depicted a scenario in which the reference color was represented by yellow circles (12 trials), the other half being represented by blue circles (12 trials). Truth-conditions were equally balanced across all trials. This experimental paradigm yields for each type of instruction probe (linguistic, non-linguistic) a total of 240 trials (2 quantified expressions * 5 ratios * 2 reference color * 2 sentence color * 6 trials), or a total of 480 trials. The linguistic and non-linguistic instruction probes were equally divided into 2 runs, yielding two linguistic runs and two non-linguistic runs. Each run was built out of a total of 120 experimental trials, equally balanced across conditions, and 75 rest trials whose presentation was optimized using Optseq2 (<https://surfer.nmr.mgh.harvard.edu/optseq/>).

*Geometric properties of the images*

The preparation of each visual display began by selecting a particular proportion (p_1_- p_5_) which dictated the values of *r*, *c*. The total number of circles in an image was *r*+*c*. Circles in each color were clustered together, to make estimation feasible, and relatively large numerosities were chosen, in order to preclude counting by participants. Circles were drawn on gray background whose RGB values were exactly midway between blue and yellow. In keeping with past practices, circle radii were systematically varied in order to preclude the estimation of total surface area in a particular color in lieu of numerical estimation. We thus ensured that circle size and total surface area of each color could not reliably serve as a guide for judgment. An iterative process created each stimulus de novo – participants never saw the same image twice. We inverted the color associated with each amount thereby creating two fully counterbalanced visual displays^4^. The resulting design had 5 blue/yellow proportions (4/16, 11/16, 16/16, 23/16, 64/16), where each proportion featured in 24 trials. These were equally divided between trials in which yellow was the reference numerosity *r*, and *c* (yellow) varied; and those trials in which yellow was *r* and blue was *c*. Further, the relation between *r* and the color mentioned in the sentence was also counterbalanced.

**Trial sequential structure**

Each trial started with the presentation of a fixation cross. For the auditory expression, the fixation cross stayed on the screen until the end of auditory sentence. For the visual expression, the fixation cross was presented for 200ms. Sentences were auditory, and differed only in the comparative part (**more**, **less**), whose duration was identical.

Our choice to present the participants with pairs of auditory language stimuli stemmed from our desire to dispense with noise-inducing eye movement during stimulus presentation, and to make the comprehension process as natural as possible. But the control, non-linguistic, stimulus pair was visual. The auditory/visual difference thus coincided with the Probe Type contrast. To avoid confounds due to this design, we steered clear from main effects. Our data analyses in both behavioral and neuroimaging domains were only based on interaction effects in which such confounds cannot manifest. To be sure, main and simple effects were tested for, and found (cf. Fig. 2 A, E, F main text). Yet the crux of our argument relies on the interaction for the reasons specified above.

Both auditory and visual expressions were 2200ms long. For the visual expression, we used a display mode that mimicked the temporal unfolding of the auditory sentence: first, the leftmost square was presented (750ms), then the inequality sign was added to the display (850ms), followed by the rightmost square (600ms). This display mode prevented participants from reading the non-linguistic expressions from right-to-left or using other visual strategies. All instruction probes – whether auditory or visual – disappeared at this point, and a brief 200ms delay followed. Next, the image containing an array of blue and yellow circles was displayed for 1100ms. The total length of a trial was 6 seconds. For half of the trials, a 1 second jitter was also introduced at the onset of the trial.

**Task**

Participants performed a speeded *Truth Value Judgment Task* by pressing the left or right mouse button. In the linguistic task, subjects were asked to decide whether the sentence with a quantified subject matched a visual array of blue and yellow circles. In the nonlinguistic task, subjects were asked to decide whether a visual display depicting a quantified expression using symbols matched a similar visual array. To minimize differences between the two tasks, the temporal structure of a trial was kept constant.

**Instructions** (given prior to entry into the magnet)

You will view a screen and hear one of the following sentences as auditory instructions:

*Less than half of the circles are blue*

*…a list of all sentences given in the experimental session*

***OR***

You will see a screen, and receive one of the four following visual instructions:


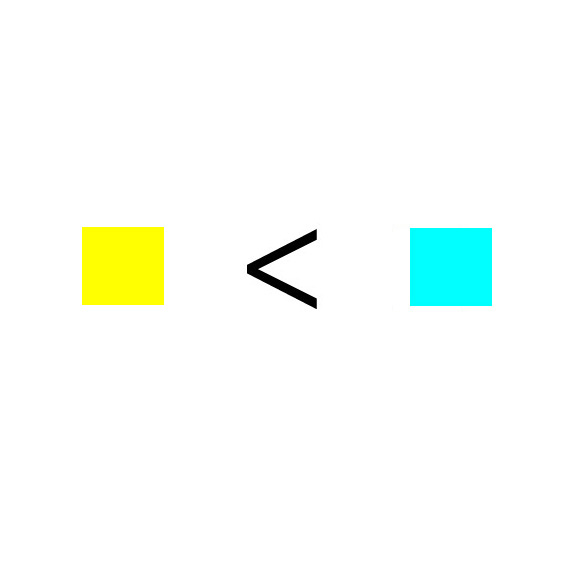


*…a list of all visual stimuli given in the experimental session*

Each symbol will be presented sequentially (in 3 steps):


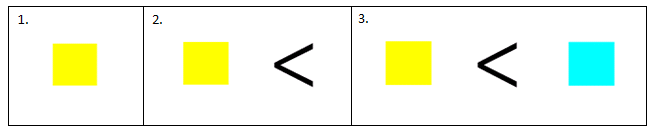


After each instruction probe, an image containing blue and yellow circles will appear on the screen. The number of circles will vary, as will their sizes.


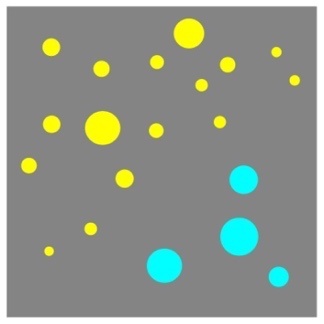

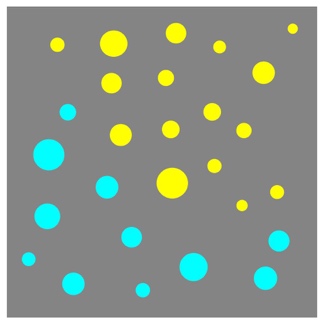


Your task is to determine whether the instruction matches the scenario in the image, and do so as quickly as you can. Press the **left button** if the sentence is **TRUE**, and the **right button** if the sentence is **FALSE**. Make sure to respond as quickly as you can. Again: **left button** if the sentence is **TRUE**, and **right button** if the sentence is **FALSE**.

**Do not try to use a ‘strategy’ to perform the task, such as counting individual circles or relying on the approximate surface area of the colors. Such strategies won’t work. Do your best to focus on your capacity to quickly estimate the number of each color of circle, and base your decisions on this.**

**fMRI methods**

The experiment was conducted at the Montreal Neurological Institute using a 3T Siemens Trio scanner. Participants wore MR-compatible headphones (Sensimetrics Corporation, Malden, MA). To reduce head motion, participant’s head was immobilized by means of a vacuum-bag filled with polystyrene balls and a forehead-restraining device (Hybex Innovations, St-Leonard, Qc, CAN). The functional data were recorded from 33 axial slices oriented parallel to the AC-PC line using a gradient-echo multi-slice EPI interleaved sequence (TE= 30ms, TR=2.0, Flip Angle 90º, matrix 64x64, FOV=224x224, slice thickness 3.5mm, isotropic, no gap). Four time-series containing 442 images each were recorded (two series of linguistic task and two series of non-linguistic task). A high-resolution T1-weighted volume was acquired for anatomical localization after two functional scans (R scripts for RT analysis, and AFNI scripts for fMRI analysis, are available at <https://www.grodzinskylab.com/data-and-code>).

**Pre-processing**

The four time-series were aligned to the anatomical scan using local Pearson correlations^6^. The times series were then spatially registered, motion-corrected (within and across runs), de-spiked, converted to a percentage of signal change and smoothed with Gaussian 6-mm FWHM filter using AFNI’s 3dBlurToFWHM^7^. All functional volumes acquired during excessive motion (i.e. >1mm) were excluded from the analyses (AFNI’s *censor* function).

**Statistical analyses**

For each trial, we modeled two different time windows: the composition and the comparison phase. Phase focuses on the presentation of the auditory or visual expression (composition phase) whereas, Phase II (comparison phase) focuses on the visual display of yellow and blue circles. Each phase (i.e. composition and comparison), was analyzed using its own event-related GLM. Note that individual event-related GLMs for each processing step were licensed since that the temporal spacing between the onset of the instruction phase (auditory sentence or quasi-algebraic expressions) and the onset of the visual display depicting blue/yellow circles was more than 1s^7^.

*Phase I: Composition mask*

The first analysis sought to identify regions that were activated during the processing of the auditory linguistic expression (less than half (LH), more than half (MH)) or the quasi-algebraic visual expression (<.>).

At the first level, for each condition (LH, MH, <, >) event-related responses were analyzed using AFNI’s 3dDeconvolve. Each stimulus was modeled using a 1-parameter block shape basis function (fixed-shape regression; AFNI model BLOCK5) with a duration of 2.6 s (corresponding to the length of instruction (both the auditory and quasi-algebraic visual instruction had the same duration)). Additional regressors for the mean, the linear and the quadratic trend components as well as the 6 motion parameters (x, y, z, roll, pitch and yaw) were also included in the model. The anatomical and functional datasets were then spatially normalized to the MNI_avg152T1 template using the 12 parameters affine transform implemented in AFNI (@auto_tlrc program). As implemented by the @auto_tlrc program, the T1 image is first normalized to the template, and subsequently the T2 images are normalized to the normalized T1 image.

Whole-brain group analyses were performed using the participants’ beta values taken from the first level analysis (AFNI program 3dANOVA3). A two-way repeated measures ANOVA on the BOLD signal, with Instruction (linguistic, non-linguistic) and Polarity (positive, negative) as within subject-factors. A cluster correction for multiple comparisons across the brain was implemented using AFNI’s 3dClustSim. Based on the results of 3dClustSim, it was determined that a family-wise error (FWE) rate of p < 0.05 is achieved with a minimum cluster size of 25 contiguous voxels each significant at p < 0.005.

*Phase II: Comparison mask*

At the first level, for each condition (LH, MH, <, >) event-related responses were analyzed using AFNI’s 3dDeconvolve. To detect brain regions where the BOLD signal was modulated by the difficulty associated with comparing the numerical distance between the number of target color circles and the non-target color circles, the average RTs for each condition was used as an amplitude-modulated parametric regressor (implemented via 3Deconvolve with *stim_times_AM2* option). Each stimulus was modeled using a 1-parameter block shape basis function (fixed-shape regression; AFNI model BLOCK5) starting at the onset of the presentation of the visual display an array of blue and yellow circles (2.6 s) and with a duration of 700ms (we used the upper end of the group reaction time excluding the 16:16 ratio). Additional regressors for the mean, the linear and the quadratic trend components as well as the 6 motion parameters (x, y, z, roll, pitch and yaw) were also included in the model. The anatomical and functional datasets were then spatially normalized to the MNI_ vg152T1 template using the 12-parameter affine transform implemented in AFNI (@auto_tlrc program). As implemented by the @auto_tlrc program, the T1 image is first normalized to the template, and then the T2 images are normalized to the normalized T1 image.

Whole-brain group analyses were performed using the participants’ beta values taken from the first level analysis (AFNI program 3dANOVA3). This analysis examined the relationship between RTs and the BOLD signal. This was done by averaging data from the linguistic and non-linguistic conditions. One statistical map was generated. A cluster correction for multiple comparisons across the brain was implemented using AFNI’s 3dClustSim. Based on the results of 3dClustSim, it was determined that a family-wise error (FWE) rate of p < 0.05 is achieved with a minimum cluster size of 25 contiguous voxels each significant at p < 0.005.

We computed the conjunction, the map of joint activation between first and second time window^8^. Only voxels that were significant at p = 0.05 (uncorrected) in both each map were included in the conjunction map. The resulting group map was corrected using the same parameters as the whole-brain group maps described above.

**Cytoarchitectonic methods**

**Histological processing**

Ten human postmortem brains (5 males, 5 females). Donors, who had no clinical history of neurological or psychiatric disease, were obtained from the body donor program of the University of Duesseldorf, in accordance to the Guidelines of the Ethics Committee.

Brains were removed from the skull 24-36 hours after death and fixed in 4% formalin or in Bodian’s fixative for 6 months or more. Prior to histological processing, MR imaging was performed on a Siemens 1.5 T scanner (Erlangen, Germany), using a T1-weighted 3D FLASH sequence (flip angle: 40º, RT: 40 ms, TE: 5 ms). These images were then used as undistorted spatial references for subsequent 3D reconstruction of the brains (for more details, see^9,10^).

Next, the brains were embedded in paraffin and serially sectioned in coronal plane (thickness: 20 µm). Block-face images of the embedded brains were acquired at every 60^th^ sections with a CCD camera (XC-75, Sony, Japan, image matrix: 256×256 pixels, 8 bit grey resolution). Every 15^th^ section was mounted on a glass and stained for cell bodies using a silver staining method^11^. Every fourth section (each 60^th^ section overall) was then analyzed for quantitative cytoarchitecture of area Id7 (Fig. 3A; Fig. 5).

**Cytoarchitecture of area Id7 and localization with respect to sulcal landmarks and neighbouring areas**

Id7 represents a six-layered dysgranular area with an inner cortical layer that is not always present when moving along the cortical mantle, since pyramidal cells from deeper layer III and upper layer V intermingle with granular cells. Layer II is poorly developed and barely separated from layer III, as the granular cells of layer II intermingle with the small pyramidal cells from upper layer III. Neurons in deeper sublayer III are mostly medium-size pyramidal cells, with the exception of few large pyramidal cells (Fig. 3A main text). Layer V can be subdivided into an upper sublayer with densely packed, large pyramidal cells, and a deeper, less densely packed sublayer. Layer VI is again more densely packed than lower sublayer V, making the border between them clear-cut. The border between layer VI and white matter is less distinct as layer VI gradually spreads into the white matter.

The area was located in the most anterior portion of the insula. Dorsally, it bordered to a yet uncharted opercular area of the inferior frontal gyrus (Op7), through the superior limiting sulcus. Op7 could be distinguished from Id7 as its layer II contains more granular cells, layer III shows higher density of pyramidal cells, and layer IV is mostly made by clusters of granular cells. In addition, layer VI displays a dense packing of multiform cells, and therefore it can be better separated from the white matter (Fig. 5G). Ventrally, Id7 borders an area at the transition of the insula to the orbitofrontal cortex, which has also not been mapped yet. It was called Area Orbito-Insularis (AOI). This area shows, in contrast to Id7, a lower density of granular cells in layer II, a decrease in density of pyramidal cells in both layer III and V, as well as a less developed layer IV (Fig. 5H).

**Cytoarchitectonic mapping and analysis of volumetric and interhemispheric differences of area Id7**

In this study, we identified and mapped a new cytoarchitectonic area, Id7 (“I” for Insular lobe, “d” for dysgranular area, “7” for the most anterior area of the insula. The area was identified in serial cell-body stained histological sections (Fig. 5) based on image analysis and statistical tools to define borders of areas^11,12^. Using an Axiovision system with a motorized scanning stage (Zeiss), each region of interest ROI was digitized and converted, by adaptive thresholding, into a gray level index (GLI) image using in house programmed applications of MatLab for Windows (MatLab R2009a; Mathworks Inc., Natick, MA, USA). In these GLI images, the value of each pixel is a measure of the volume fraction of cell bodies in the corresponding measuring field^12^. Next, an outer border between layer I and II and inner border between layer VI and white matter were interactively traced on the GLI images. GLI profiles were extracted along curvilinear, parallel transverses, running from the outer to the inner contour line, perpendicularly to the cortical layers (Fig. 5D).

The profiles shape was quantified by a 10-dimensional feature vector, including mean GLI, cortical depth of the center of gravity, standard deviation, skewness, kurtosis of the profile, and the corresponding parameters of the first derivative of the profile. The differences between feature vectors identified differences in profiles shape and, therefore, in cytoarchitecture, and were measured using the Mahalanobis distance, MD^12,13^. A cytoarchitectonic border was detected at the position where the MD reached a significant (p˂0.05) maximum, according to Hotelling’s T^2^–test with Bonferroni correction for multiple comparisons. To increase the signal to noise ratio, the MD were calculated in adjacent blocks of profiles (each consisting of 8 to 24 GLI profiles), which move as a sliding window along the cortical ribbon, in steps of one GLI profile increment^12,13^ (Fig. 5E-H). Borders of area Id7 were then labeled in images of the histological sections and 3D-reconstructed.

The volumes of left and right Id7 were stereologically estimated as previously described^14^. Depending on the extent of area Id7, 7-10 histological sections were analyzed per hemisphere and brain. Mean volume and standard deviation were calculated for each hemisphere. Volumes did not differ between hemispheres according to a paired *t*-test (p>0.05). It was larger on the left than on the right in 6 out of ten brains, and larger on the right than on the left in 4 brains. The volumes varied between brains and ranged from 197 mm^3^ (brain nº7, left hemisphere) to 672 mm^3^ (brain nº10, right hemisphere).

To check for putative left-right differences in cytoarchitecture, groups of 15 GLI profiles were extracted from random sections of area Id7 in each hemisphere, resulting in a total of 900 GLI profiles (45 profiles per hemisphere, 20 hemispheres), and feature vectors were extracted to describe their shape, i.e., cytoarchitecture.  Interhemispheric differences in the shape profiles were analyzed using an ANOVA, with “area” and “hemisphere” as factors, and “brain” as blocking factor (Systat^®^ 13 for Windows), but did not reach significance (p>0.05).

**Probabilistic cytoarchitectonic maps**

To generate 3D reconstructions of the histological sections (Fig. 3 main text), we used the block-face images of the paraffin-embedded brains, and the T1-weighted MR scans of the fixed brains taken prior to histological processing to correct for deformation due to histological processing^9,10^. All 3D-reconstructed data-sets were normalized to the individual T1-weighted single-subject template of the MNI space^15,16^, using a combination of linear transformation, grey level normalization and non-linear elastic registration algorithms^10^. Probability maps were generated by superimposition the individual areas in this space. The degree of overlap among the ten individual areas was described by values from 0 to 100%, and color coded (Fig. 3C main text). Id7 probability maps showed large regions of overlap (i.e., low intersubject variability) in both hemispheres, and relatively small regions with low overlap (and higher intersubject variability) in the periphery (Fig. 3C main text).

Binarized versions of the probabilistic anatomical maps were used for the fMRI analysis and their 3D visualization. Therefore, the probabilistic maps were thresholded at the level 40 %. I.e., the binarized maps comprised only those voxels, which belonged to the anatomical region in at least 4 post-mortem brains. The threshold was chosen such that the volume of the binarized map was as close as possible to the average volume of the individual maps.

The cytoarchitectonic probabilistic map is available through the JuBrain atlas, and the HBP Human Brain Atlas (<https://www.humanbrainproject.eu/en/explore-the-brain/atlases/>).


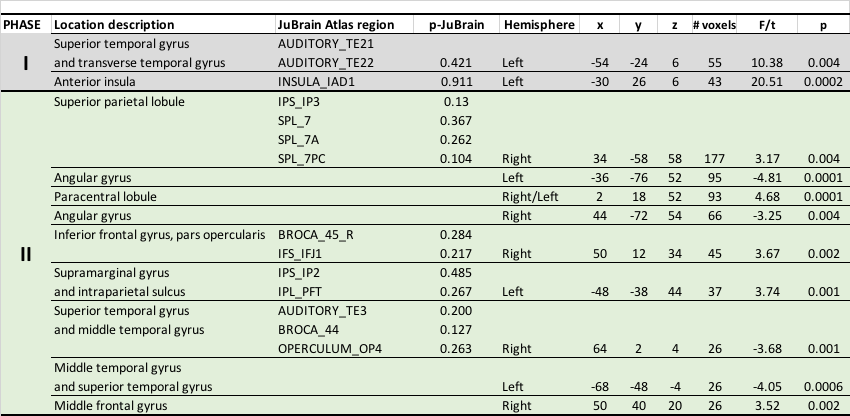
Cluster overlap calculation

The overlap of a cluster in the fMRI map and the anatomical maps was calculated as follows: Firstly, the statistical maps resulting from the fMRI analysis were binarized at a threshold of p=0.005 with a minimum cluster size of 25 voxels, yielding a map that contained two clusters. These maps were resampled to the MNI space at a resolution of 1 mm in each spatial direction, and superimposed with the cytoarchitectonic probabilistic maps in the same reference space, as the sum of probabilistic values *p_R_(x)* over the cluster *C*, divided the sum of *p_R_(x)* over the whole region:

$$Overlap\left( R, C \right)= \frac{\sum_{x\in C} p_{R}\left( x \right)}{\sum_{x} p_{R}\left( x \right)}$$

(with $p_{R}\left( x \right)=$ probabilistic value of a region *R* at voxel location *x*).

**3D-Visualization of data**

The open-source software *ParaView* ([www.paraview.org](http://www.paraview.org/); Kitware Inc., New York, USA) was used for the 3-dimensional visualization of the fMRI activation clusters and cytoarchitectonic maps (as shown in Fig. 3 main text). The probabilistic anatomical maps were thresholded as described above.

**Movie S1 caption: Anatomical region and functional activation in logical processing task.**

An expansion of Fig. 6: the visualization here is a three-dimensional reconstruction of a brain: the left frontal lobe is gradually removed to show the spatial relations between the fMRI activation cluster (magenta) and the cytoanatomically defined region Id7 (blue), as well as areas 44 (red) and 45 (yellow) in Broca's region. The blue and red dots indicate the centres-of-mass of region Id7 and the fMRI cluster, respectively. The functional activation cluster and area Id7 overlap to a large extent, and are clearly separated from areas in Broca's region. Op7, Opercular area 7; Id7, Insular dysgranular area 7; AOI, Area Orbito-Insularis; BA44, Brodmann’s Area 44; BA45, Brodmann’s Area 45.

**References:**

1 Hackl, M. *Comparative quantifiers* PhD thesis thesis, MIT, (2000).

2 Heim, I. in *Proceedings of SALT X.* (eds B. Jackson & T. Matthews) 40-64 (CLC Publication).

3 Just, M. A. & Carpenter, P. A. Comprehension of negation with quantification. *Journal of Verbal Learning and Verbal Behavior* **10**, 244-253, doi:http://dx.doi.org/10.1016/S0022-5371(71)80051-8 (1971).

4 Deschamps, I., Agmon, G., Loewenstein, Y. & Grodzinsky, Y. The processing of polar quantifiers, and numerosity perception. *Cognition* **143**, 115-128, doi:10.1016/j.cognition.2015.06.006 (2015).

5 Oldfield, R. C. The assessment and analysis of handedness: the Edinburgh inventory. *Neuropsychologia* **9**, 97-113 (1971).

6 Saad, Z. S. *et al.* A new method for improving functional-to-structural MRI alignment using local Pearson correlation. *NeuroImage* **44**, 839-848, doi:10.1016/j.neuroimage.2008.09.037 (2009).

7 Cox, R. W. AFNI: software for analysis and visualization of functional magnetic resonance neuroimages. *Comput Biomed Res* **29**, 162-173 (1996).

8 Nichols, T., Brett, M., Andersson, J., Wager, T. & Poline, J. B. Valid conjunction inference with the minimum statistic. *NeuroImage* **25**, 653-660 (2005).

9 Amunts, K. *et al.* Broca's region revisited: cytoarchitecture and intersubject variability. *J Comp Neurol* **412**, 319-341 (1999).

10 Amunts, K. *et al.* Analysis of neural mechanisms underlying verbal fluency in cytoarchitectonically defined stereotaxic space--the roles of Brodmann areas 44 and 45. *NeuroImage* **22**, 42-56, doi:10.1016/j.neuroimage.2003.12.031 (2004).

11 Merker, B. Silver staining of cell bodies by means of physical development. *J Neurosci Methods* **9**, 235-241 (1983).

12 Schleicher, A., Amunts, K., Geyer, S., Morosan, P. & Zilles, K. Observer-independent method for microstructural parcellation of cerebral cortex: A quantitative approach to cytoarchitectonics. *NeuroImage* **9**, 165-177, doi:10.1006/nimg.1998.0385 (1999).

13 Schleicher, A. *et al.* Quantitative architectural analysis: a new approach to cortical mapping. *Anat Embryol (Berl)* **210**, 373-386, doi:10.1007/s00429-005-0028-2 (2005).

14 Amunts, K. *et al.* Gender-specific left-right asymmetries in human visual cortex. *The Journal of neuroscience : the official journal of the Society for Neuroscience* **27**, 1356-1364, doi:10.1523/JNEUROSCI.4753-06.2007 (2007).

15 Evans, A. C. *et al.* Anatomical mapping of functional activation in stereotactic coordinate space. *NeuroImage* **1**, 43-53 (1992).

16 Evans, A. C., Janke, A. L., Collins, D. L. & Baillet, S. Brain templates and atlases. *NeuroImage* **62**, 911-922, doi:10.1016/j.neuroimage.2012.01.024 (2012).
